# Chest compressions quality during sudden cardiac arrest scenario performed in virtual reality: A crossover study in a training environment

**DOI:** 10.1097/MD.0000000000023374

**Published:** 2020-11-25

**Authors:** Filip Jaskiewicz, Dawid Kowalewski, Katarzyna Starosta, Marcin Cierniak, Dariusz Timler

**Affiliations:** Emergency Medicine and Disaster Medicine Department, Medical University of Lodz, Poland.

**Keywords:** cardiopulmonary resuscitation, chest compressions, virtual reality

## Abstract

Potential attributes of virtual reality (VR) can be a breakthrough in the improvement of sudden cardiac arrest (SCA) training. However, interference with the virtual world is associated with the need of placing additional equipment on the trainee's body. The primary aim of the study was to evaluate if it does not affect the quality of chest compressions (CCs).

91 voluntarily included in the study medical students participated twice in the scenario of SCA – Traditional Scenario (TS) and Virtual Reality Scenario (VRS). In both cases two minutes of resuscitation was performed.

If VRS was the first scenario there were significant differences in CCs depth (VRS - Me = 47 mm [IQR 43 – 52] vs TS - Me = 48 mm [IQR 43 – 55]; *P* = .02) and chest relaxation (VRS - Me = 37% [IQR 5 – 91] vs TS - Me = 97% [IQR 87 – 100]; *P* < .001). 97.8% of respondents believe that training with the use of VR is more effective than a traditional method (*P* < .01). Most of the study group (91%, *P* < .01) denied any negative symptoms during the VR scenario.

Virtual reality can be a safe and highly valued by medical students, method of hands-on CPR training. However additional VR equipment placed on the trainee's body may cause chest compressions harder to provide. If it is not preceded by traditional training, the use of VR may have an adverse impact on depth and full chest relaxation during the training. To make the best use of all the potential that virtual reality offers, future studies should focus on finding the most effective way to combine VR with traditional skill training in CPR courses curriculum.

## 1 Introduction

In recent years the impact of technological progress has been more and more visible in every area of life. This process can also be very noticeable in clinical medicine, health science and modern medical education. High fidelity simulation (HFS) has been one of the most promoted training methods introduced in the last two decades.^[1,2]^ HFS is based on creating conditions as close as possible to real life situation. Among teaching tools that can be used in HFS, virtual reality (VR) and augmented reality (AR) are probably the most innovative.^[3]^ VR and AR applications are already used in surgery, diagnostics, pain management, dentistry, ophthalmology and psychotherapy.^[4–8]^ The great advantage of VR is that it allows simultaneous training of practical skills, soft skills, stress and critical situation management.^[9–11]^ Potentially these attributes can be a breakthrough in the improvement of sudden cardiac arrest (SCA) recognition and management. Despite widespread training in cardiopulmonary resuscitation (CPR) and defibrillation, SCA is one of a leading cause of death in Europe with an overall resuscitation success rate less than 10%.^[12]^ From this perspective, the improvement of CPR education outcomes appears to be essential. The form of VR implementation in CPR training is very diverse. Starting from applications available for mobile phones to prototypes that allow to provide hands-on training with realistic haptic feedback.^[9,13–16]^ However, interference with the virtual world is associated with the need of placing sensors on the trainee's body (goggles and wrist bands or gloves). As it is well established that bystander high quality chest compressions (CCs) is one of the main determinants of survival in SCA,^[12,17]^ it is very important to assess if the use of additional VR equipment does not affect the quality of CCs. The importance and innovative nature of the matter prompted us to take an interest in assessment of CCs quality during SCA scenario performed with the use of VR prototype.

## 2 Objective

The primary aim of the study was to assess CCs quality during sudden cardiac arrest scenario performed with the use of VR prototype.

The secondary aims were to assess if additional equipment may cause CCs subjectively harder to provide and analyze impressions of medical students on the potential usefulness of VR CPR training prototype.

## 3 Methods

### 3.1 Study design and setting

A cross-sectional design was used in this study. Data collection was conducted at the Emergency Medicine and Disaster Medicine Department of Medical University in Lodz (Poland) and lasted from 01/04/2019 to 31/05/2019. The study received a positive opinion of the Bioethics Committee of the Medical University of Lodz (decision number: RNN/104/18/KE).

### 3.2 Participants

**Figure 1:**
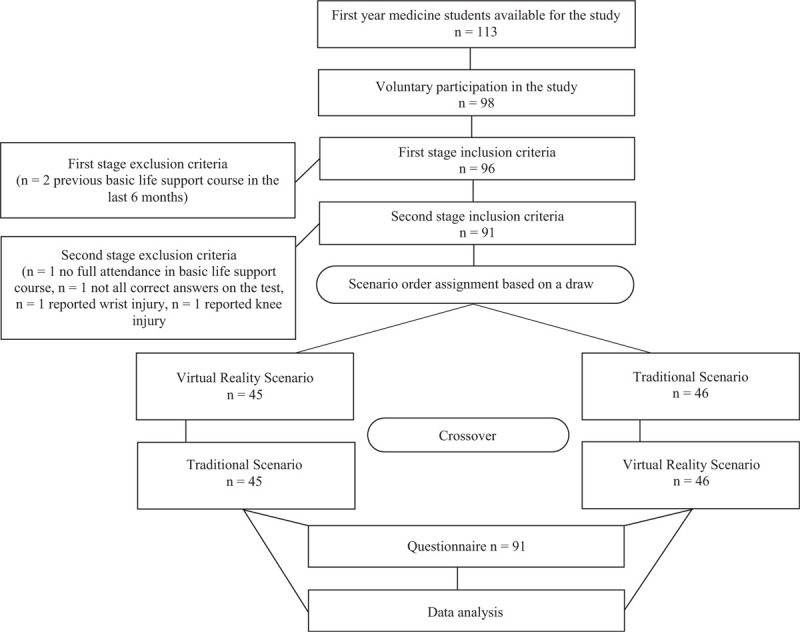
Study protocol presentation.

One hundred and thirteen students had the opportunity to take part in the study. Participation was voluntary. The study was divided into two stages (Fig. 1). Inclusion criteria to the first stage was: no previous, additional basic life support (BLS) training in the last six months. In first stage, to unify knowledge and skills in resuscitation among all participants, they have attended a three-hour BLS course - 45 minutes of theoretical background and 90 minutes of practice on the CPR mannequin (no real-time feedback concerning CCs quality was available). Program was based on European Resuscitation Council (ERC) Guidelines 2015. At the end of the course students took the multiple-response test with one correct answer. Inclusion criteria to the second stage were: full attendance in BLS course, all correct answers on the test, no reported spine, wrist or knee injury for one month before the study. On the second stage, students participated twice in the scenario of SCA – Traditional Scenario (TS) and Virtual Reality Scenario (VRS). In both cases two minutes of hands-only CPR was performed. Participants were divided into two study groups (Fig. 1). Group assignment was based on a draw.

### 3.3 Data sources/measurement

CCs were conducted on CPR mannequin — Resusci Anne QCPR with standard 50 kg compression spring and CCs quality was measured with SimPad equipped with Skillreporter software (Laerdal, Stavanger, Norway). Minimum of a 10-minute rest between exercises was obligatory. At the end of the second stage of the study each student filled out a questionnaire consisting of five questions regarding to previous experience with VR and impressions on the applicability of VR in the resuscitation training. VRS was conducted with the use of VR prototype (beta version of CPR virtual reality learning software – VR ACT, Octopus VR, Lodz, Poland). The VR ACT software was implemented for HTC Vive (HTC, Taoyuan, Taiwan) and Noitom Hi5 VR Glove (Noitom International Inc. Miami, Florida, USA). In VRS participants practiced on the same CPR mannequin but it was integrated in the virtual space and covered with virtual 3D-human model.

### 3.4 Statistical analysis

Statistical analysis of the data was carried out using PQStat ver. 1.6.8.228 (PQStat Software 2020, Poznan, Poland). Quantitative variables are presented using basic descriptive statistics: the arithmetic mean (x), standard deviation (SD), median (Me) and interquartile range [IQR] or percentages (%). The results of the analyzed VRS and TS CCs measurement were compared by the Wilcoxon pair order test. A nonparametric approach to analysis was chosen after prior analysis of distribution normality using the Shapiro-Wilk test. Analysis of the CCs results depending on the order of the scenario was made with the use of Mann-Whitney U test. Comparison of the answer distribution on survey questions to the assumed level of 50% was carried out with the Z test for one proportion. Test probability at p < .05 was considered as significant and test probability at *P* < .01 was considered as highly significant.

## 4 Results

Out of 113 first year medicine students, 98 voluntarily participated in the study. Ninety-one met the inclusion criteria to the second stage (Fig. 1). Mean age was 19.8 ± 1.3 years. Body mass index (BMI) was calculated for all students: 21.6 ± 2.9 (Me = 21.3 [IQR 19.5 – 23.5]). Most participants did not have previous experience with VR, but the difference was not statistically important (respectively 59.3% vs 40.7%; 95% CI 0.48 – 0.69; p = .09). Only 11% of participants had previous contact with VR in form which gave the possibility of interaction with the virtual world. In general analysis there were no statistically important differences between VRS and TS groups neither in CCs rate nor in CCs depth (Table 1.). A highly statistically significant difference was found in percentage of full chest relaxation (FCR) component (respectively for VRS group Me = 69% [IQR 26 – 98] vs TS group Me = 97% [IQR 85 – 100]; p < .001). An additional analyzes were performed depending on the order of the scenario. In the component of CCs rate, the results were significantly higher in each first scenario, regardless of whether it was performed as VRS or TS (Table 2.). CCs depth was significantly lower in VRS group (Table 3.) if VRS was the first scenario (respectively for VRS group Me = 47 mm [IQR 43 – 52] vs TS group Me = 48 mm [IQR 43 – 55]; *P* = .02). Finally the comparison of FCR component demonstrated significantly lower percentage of relaxation (Table 4.) if VRS was the first scenario (respectively for VRS group Me = 37% [IQR 5 – 91] vs TS group Me = 97% [IQR 87 – 100]; *P* < .001). The measurement system did not note any breaks in CPR during the 2 minutes of any TS or VRS sessions.

**Table 1:** Comparison of the chest compressions quality based on rate, depth and full chest relaxation measurements in Virtual Reality Scenario and Traditional Scenario groups.

n = 91	x	SD	Me	IQR	Z value	*P* value
TS CCs rate (per minute)	114.9	11.8	114	108–122	0.701	.48
VRS CCs rate (per minute)	114.2	11.9	115	108–122		
TS CCs depth (mm)	49.3	7.4	48	44–55	1.886	>.05
VRS CCs depth (mm)	47.8	8.8	49	43–53		
TS CCs relaxation (%)	83.7	27.9	97	85–100	4.627	<.01
VRS CCs relaxation (%)	60.2	37.5	69	26–98		

**Table 2:** Comparison of the chest compressions rate in Virtual Reality Scenario and Traditional Scenario groups depending on the scenario order.

	VRS CCs rate (per minute)				TS CCs rate (per minute)				
Scenario order	x	SD	Me	IQR	x	SD	Me	IQR	*P* value
TS - VRS	112.7	11.3	113.0	106.5–120.8	118.7	12.6	115.0	108.3–129.8	.001
VRS - TS	115.7	12.6	117.0	109–122	111.0	9.6	113.0	105–118	.04
P value	.33				.02				

**Table 3:** Comparison of the chest compressions depth in Virtual Reality Scenario and Traditional Scenario groups depending on the scenario order.

	VRS CCs depth (mm)				TS CCs rate (mm)				
Scenario order	x	SD	Me	IQR	x	SD	Me	IQR	*P* value
TS - VRS	48.0	9.6	50.0	43.5–57.7	49.4	8.4	48.0	45.2–56.7	.50
VRS - TS	47.5	8.0	47.0	43-52	49.2	6.4	48.0	43-55	.02
P value	0.58				0.49				

**Table 4:** Comparison of the full chest relaxation percentage in Virtual Reality Scenario and Traditional Scenario groups depending on the scenario order.

	VRS CCs relaxation (%)				TS CCs relaxation (%)				
Scenario order	x	SD	Me	IQR	x	SD	Me	IQR	*P* value
TS - VRS	74.7	30.4	92.0	58.3–99	79.5	32.7	95.0	69–100	.37
VRS - TS	45.4	38.6	37.0	5–91	88.0	21.4	97.0	87–100	<.001
P value	<.001				.40				

In the questionnaire which was filled out at the end of the second stage of the study the vast majority of participants (97.8%, 95% CI 0.92 – 0.99; *P* < .01) rated the educational value of VR in CPR training at 3 or 4 points in 1 to 4 scale (defined as: 1 – very low, 2 - low, 3 – high and 4 very high). The same 97.8% of respondents believe that resuscitation training with the use of VR is more effective than a traditional method (*P* < .01). Statistically important majority of the study group (76%, n = 69, 95% CI 0.65 – 0.84; *P* < .01) stated that additional VR equipment (goggles and gloves) made CCs subjectively more difficult to perform. Yet it is worth noticing that on four-point scale (where additional difficulties were defined as: 1 - none, 2 - minor, 3 - significant and 4 very significant), majority of the study group (80%, n = 73, 95% CI 0.70 – 0.87; *P* < .01) rated difficulties as none (n = 23) or minor (n = 53). Still 20% of participants (n = 18) rated troubles with CCs performance at 3 or 4 points. The questionnaire also assessed whether students felt any negative physical symptoms during the VR scenario. Most of the study group denied (respectively 91% of respondents, 95% CI 0.83 – 0.96; *P* < .01). The remaining 9% of students reported: blurred vision caused by goggles use (n = 4) or fatigue (n = 4).

## 5 Discussion

As in most communities the median time from emergency call to ambulance arrival is longer than the period of brain cells ability to live without oxygen. The immediate bystander CPR is crucial for SCA victims. It can double or quadruple survival rate chances.^[12,17]^ International guidelines indicate that key elements of CPR training are the ability to recognize SCA and readiness to undertake CPR by witnesses.^[12,17]^ VR or AR capability of simulating different surroundings of the event scene, providing an interaction with bystanders and presenting of SCA symptoms (e.g. gasping, cyanosis) can have a positive impact on education.^[9–11]^ Traditional CPR training does not provide any emotional realism to the performance of skills while immersive VR is able to stimulate physiological, psychological and social responses from participants similar to those in the real world.^[11]^ In Buckler et al. study, participation in a VR simulated SCA event was associated with increased self-reported confidence in all domains of bystanders interventions.^[11]^ The increased feeling of responsibility reported by study group in Buckler et al. research, highlights the potential benefits of combining VR-based skills scenarios into traditional CPR training.^[11]^ The opinion of the participants about the use of VR in CPR training on the presented prototype was very positive and consistent with the reports of other authors.^[15,16,18]^ On a four-point scale, 97.8% of them rated its educational value as high or very high (*P* < .01). The same 97.8% of respondents believe that resuscitation training with the use of VR is more effective than traditional method (*P* < .01). Importantly, most of the study group denied feeling any negative physical symptoms during VR scenario (91%, n = 83; *P* < .01). Those reported by 8 students (blurred vision or fatigue) probably are not a threat to health and may be naturally caused by using VR goggles and physical effort of CPR itself. As in the present study, VR prototype enabled hands-on CPR training the primary aim was to assess CCs quality during VR scenario. Additional equipment including VR goggles and gloves may potentially cause CCs harder to provide. CCs quality is important factor affecting brain perfusion and potential return of spontaneous circulation (ROSC). According to the international guidelines, the quality of CCs is influenced by four parameters: rate, depth, degree of full chest relaxation and minimization of breaks in compressions. Considering that the low quality of CCs is associated with a lower survival rate, all courses should focus on these elements.^[12,17]^ In the present study there was no statistically important difference in CCs rate between VRS and TS groups (*P* > .05). The results of the analysis depending on the order of the scenarios showed differences between the VRS and TS groups but CCs rate results were still within the range of 100–120 per min, which is recommended by the guidelines.^[12,17]^ In general analysis CCs depth also did not differ between study groups. Participants did not reach the minimum value of 50 mm recommended by the guidelines but obtained results do not differ from those presented in other studies conducted in training conditions.^[19–23]^ It is worth noticing that CCs depth was significantly lower in VRS group when VRS was the first scenario. Highly statistically important difference was also found in in percentage of FCR component (*P* < .01). It is interesting that such a tendency was found in the general analysis and in the variant when the VRS was the first training session (*P* < .01), but there was no important difference in FCR percentage between study groups if VRS was applied as a second scenario (respectively for VRS group Me = 92% [IQR 58.3 – 99] vs TS group Me = 95% [IQR 69 – 100]; *P* > .05). After reaching correct CCs depth, FCR results in the creation of an appropriate pressure difference in the chest. That determines the perfusion pressure and makes FCR an important CPR quality factor. Incomplete chest recoil will result in a reduction of perfusion pressure, thereby reducing chances of ROSC.^[12,17,23–27]^ FCR is the most difficult part for participants to perform within such a short time especially for those training in CPR for the first time.^[28]^ The results of other studies conducted in the training environment regarding the percentage of FCR are very diverse and difficult to compare with those obtained in the present study due to methodological differences.^[20,24,28]^ The reason why the difference in FCR between the TS group and VRS was so significant is unclear, but it is worth to notice that majority of students stated that, additional VR equipment (goggles and gloves) caused some difficulties in CCs performance (76%, n = 69; *P* < .01). Most of them (80%, n = 73; *P* < .01) rated difficulties as none or minor but for one fifth of respondents additional equipment makes CCs significantly and very significantly harder to perform in comparison to traditional training. However, this does not explain the fact that when VRS was the second scenario, there were no statistically significant differences between the TS group and VRS neither in CCs depth nor in FCR percentage. As it is possible to use various types of VR hardware, it will be necessary to assess the effect of individual technical and technological solutions on CCs quality and users’ comfort in future research. Virtual reality can be a safe and highly valued by medical students, method of hands-on CPR training. However additional VR equipment placed on the trainee's body may cause CCs harder to provide. If it is not preceded by traditional training, the use of VR may have an adverse impact on depth and FCR during the training. To make the best use of all the potential that virtual reality offers, future studies should focus on finding the most effective way to combine VR with traditional skill training in CPR courses curriculum.

## 6 Limitations of the study

The study was conducted only on one specific VR prototype. In future studies CCs quality should be assessed considering also other technological solutions. Every participant took part only in single, two-minute VR scenario. Future studies should take into consideration an assessment of full training models, scenario order and the use of real-time feedback devices.

## Author contributions

**Conceptualization:** Filip Jaskiewicz.

**Data curation:** Filip Jaskiewicz, Dawid Kowalewski, Katarzyna Starosta, Marcin Cierniak.

**Formal analysis:** Filip Jaskiewicz, Dariusz Timler.

**Investigation:** Filip Jaskiewicz, Dawid Kowalewski, Katarzyna Starosta, Marcin Cierniak.

**Methodology:** Filip Jaskiewicz.

**Project administration:** Filip Jaskiewicz.

**Supervision:** Filip Jaskiewicz.

**Writing – original draft:** Filip Jaskiewicz.

**Writing – review & editing:** Filip Jaskiewicz.
